# Treatment of Chronic Myeloid Leukemia in Rural Rwanda: Promising Early Outcomes

**DOI:** 10.1200/JGO.2015.001727

**Published:** 2016-02-03

**Authors:** Neo Tapela, Ignace Nzayisenga, Roshan Sethi, Jean Bosco Bigirimana, Hamissy Habineza, Vedaste Hategekimana, Nicholas Mantini, Tharcisse Mpunga, Lawrence N. Shulman, Leslie Lehmann

**Affiliations:** **Neo Tapela, Roshan Sethi,** and **Nicholas Mantini,** Brigham and Women’s Hospital; **Neo Tapela, Roshan Sethi, Lawrence N. Shulman,** and **Leslie Lehmann,** Harvard Medical School; **Leslie Lehmann,** Children’s Hospital of Boston, Boston, MA; **Neo Tapela, Ignace Nzayisenga, Jean Bosco Bigirimana, Hamissy Habineza, Nicholas Mantini, Lawrence N. Shulman,** and **Leslie Lehmann,** Partners In Health/Inshuti Mu Buzima; **Vedaste Hategekimana** and **Tharcisse Mpunga,** Ministry of Health, Kigali, Rwanda; and **Lawrence N. Shulman,** University of Pennsylvania, Philadelphia, PA.

## Abstract

**Purpose:**

The burden of cancer is rising in low- and middle-income countries, yet cancer treatment requires resources that are often not available in these settings. Although management of chronic myeloid leukemia (CML) has been described in low- and middle-income countries, few programs involve patients treated in rural settings. We describe characteristics and early outcomes of patients treated for CML at rural district hospitals in Rwanda.

**Methods:**

We conducted a retrospective review of patients with confirmed BCR-ABL–positive CML who were enrolled between July 1, 2009 and June 30, 2014. Types of data included patient demographics, diagnostic work up, treatment, clinical examination, laboratory testing, and death.

**Results:**

Forty-three patients were included, with a maximum follow-up of 58 months. Of 31 patients who were imatinib-naïve at enrollment, 54.8% were men and the median age at diagnosis was 36.9 years (interquartile range: 29-42 years). Approximately two-thirds of patients (67.7%) were on the national public insurance scheme. The imatinib dose was reduced for 16 patients and discontinued for five. Thirty-two of the 43 patients continued to have normal blood counts at last follow-up. Four patients have died and four are lost to follow-up.

**Conclusion:**

Our experience indicates that CML can be effectively managed in a resource-constrained rural setting, despite limited availability of on-site diagnostic resources or specialty oncology personnel. The importance of model public-private partnerships as a strategy to bring high-cost, life-saving treatment to people who do not have the ability to pay is also highlighted.

## INTRODUCTION

Cancer accounts for an increasingly significant burden of disease in low- and middle-income countries (LMICs), where nearly two-thirds of cancer-related deaths occur.^1-3^ Unfortunately, the delivery of cancer care is complex and resource intensive. Multiple treatment modalities and multidisciplinary expertise are required, including histopathology diagnostic capacity, oncology-trained specialists, radiotherapy centers, and funds for expensive medications. Resource constraints contribute to the inequities in cancer outcomes between LMIC and high-income countries.^1^

Chronic myeloid leukemia (CML) is a hematologic malignancy that can affect patients of all ages. It is unique in that a daily oral medication, imatinib, can result in long-term disease control with good quality of life. The diagnosis of CML is suggested by splenomegaly and an elevated peripheral WBC count with immature WBCs. The diagnosis is confirmed by molecular testing for the BCR-ABL fusion protein that results from the t(9;22) chromosomal translocation. It is this fusion protein that serves as the substrate for imatinib, which is a tyrosine kinase inhibitor. Although molecular testing for BCR-ABL is readily available in the developed world, it is rarely available in LMIC.

CML is highly responsive to imatinib, as established by the International Randomized Study of Interferon and STI571 (IRIS).^4^ Compared with combination therapy of interferon and cytarabine, imatinib achieved superior hematologic and cytogenetic response, freedom from disease progression, and lower toxicity at 18 months of follow-up.^4,5^ At a median follow-up of 60 months, the estimated overall survival (OS) rate for patients initially treated with imatinib was 89%.^4^

Unfortunately, imatinib is a costly drug and thus unaffordable for many. Since 2002, imatinib has been made available for free to patients in resource-constrained settings through the Gleevec International Patient Assistance Program (GIPAP).^6,7^ All patients are required to have confirmation of the BCR-ABL translocation in their leukemia cells. GIPAP assesses treatment programs for their ability to appropriately treat and monitor patients to ensure safe and effective drug administration. In 2007, there were 18,004 patients with CML worldwide who were being treated with the assistance of GIPAP. However, only 6% (1,021) of those patients lived in Africa.^6^ With GIPAP’s support, a handful of programs have reported successful treatment of CML in resource-constrained settings.^6,8-17^ However, most of these programs are based in private and/or urban academic facilities; there are few examples of programs for patients treated in rural settings.

Since 2008, patients with CML have been diagnosed and treated in two rural Ministry of Health district hospitals in Rwanda. This was achieved through the support of GIPAP, the nongovernmental organization Partners In Health, and oncology experts from Dana-Farber/Brigham and Women’s Cancer Center (DF/BWCC, Boston, MA). In this study, we describe the management and outcomes of patients with CML receiving therapy in this public-private program.

## METHODS

### Study Setting

The program was implemented at two public district hospitals in rural Rwanda, operated by the Ministry of Health in close collaboration with Partners In Health. Rwinkwavu Hospital, a 140-bed facility serving a catchment area of 207,757 people in eastern Rwanda, became the country’s first GIPAP-registered facility in 2008. Butaro Hospital is a 152-bed facility serving 321,000 people in the Northern Province of Rwanda. The Butaro Cancer Center of Excellence, which opened in 2012, serves as a national referral center for cancer care and receives patients from several neighboring countries.^18,19^ Both hospitals anchor integrated care delivery systems comprising a district-wide network of health centers and several thousand community health workers (CHWs).

### Patient Management

Patient care at both facilities was directed by trained generalist physicians and nurses, in consultation with oncology specialists at DF/BWCC. Patients suspected of having CML underwent evaluation including detailed medical history and examination, CBC with differential, peripheral blood smear review, bone marrow aspirate, and BCR-ABL testing. Diagnostic bone marrow biopsy was not performed in the majority of earlier cases because of resource limitations, but over time has become incorporated into standard care. BCR-ABL testing was not routinely available within the country; specimens were sent to DF/BWCC for pro bono molecular diagnosis. A few patients, particularly those from neighboring countries, were diagnosed through pathology performed outside of Rwanda. Multiple diagnostic methods were used over time. Of the 35 patients with adequate documentation on type of diagnosis available at the time of manuscript submission, 33 were diagnosed via polymerase chain reaction and two by fluorescent in situ hybridization.

On initial presentation, patients were admitted to the district hospital for cytoreductive therapy with hydroxyurea and supportive management including allopurinol, intravenous fluids, and monitoring of electrolytes and renal function. Once clinically stable with a leukocyte count below 50,000 cells/μL, patients were discharged and referred for outpatient follow-up at a nurse-led integrated noncommunicable diseases clinic at the district hospital.^20,21^ Once BCR-ABL–positive status was confirmed, patients were started on imatinib 400 mg per day. Imatinib was ordered through GIPAP on a quarterly basis, at which time patients who had a confirmed diagnosis were registered. Patients were initially scheduled for follow-up every 1 to 2 months, with the imatinib dose adjusted on the basis of CBC monitoring and review of patient-reported adverse effects (Box 1).

**Box 1. Parameters for Imatinib Dose Adjustment**

**Indications for Imatinib Dose Reduction**

- Absolute neutrophil count (ANC) less than 1,000/μL, or
- Platelets less than 100,000/μL, or
- Elevated liver function tests (LFTs), however, not more than twice the normal range.

**Indications for Imatinib Discontinuation**

- ANC less than 750/μL, or
- Platelets less than 70,000/μL, or
- LFTs elevated beyond twice the normal range.

Imatinib to be reintroduced when ANC rebounded to greater than 1,000/μL, platelets greater than 75,000/μL^3^, and LFTs normalized.

In the majority of cases, follow-up molecular testing and bone marrow biopsy were not performed due to limited resources. Patients who were asymptomatic and in complete hematologic remission (CHR), as defined below, were scheduled for visits every 3 to 4 months. Socioeconomic supports, including nutritional supplementation and transport vouchers, were available for patients in need. CHW accompaniment was offered to vulnerable patients residing within the hospitals’ catchment districts.

### Data Collection

We conducted a retrospective review of patients with confirmed BCR-ABL–positive CML who were enrolled between July 1, 2009 and June 30, 2014. Manual chart abstraction was performed twice by independent observers at each site, with discrepancies resolved by a third observer. Data included baseline patient demographics, diagnostic work up, treatment, clinical examination, and laboratory testing at 3, 6, and 12 months (± 30 days for all time points) of follow-up. Only clinical and laboratory examinations that fell within these date ranges were included. If there were multiple visits within each range, the most complete examination was included in analysis.

Patients with incomplete documentation of BCR-ABL status were excluded from the analysis. Patients who had been diagnosed and/or who initiated imatinib treatment before establishing care at Rwinkwavu or Butaro hospitals were excluded from the baseline characteristics analysis (Table 1), but were included in all other analyses. Patients who were lost to follow-up (LTFU), as defined below, were included in survival analysis. The follow-up period was counted from the date of imatinib initiation at Rwinkwavu or Butaro hospital (even if the patient had received imatinib before establishing care there), until the last follow-up visit. Telephone calls with patients, or visits on behalf of patients by family members or CHWs, were not recorded as follow-up visits.

**Table 1:**
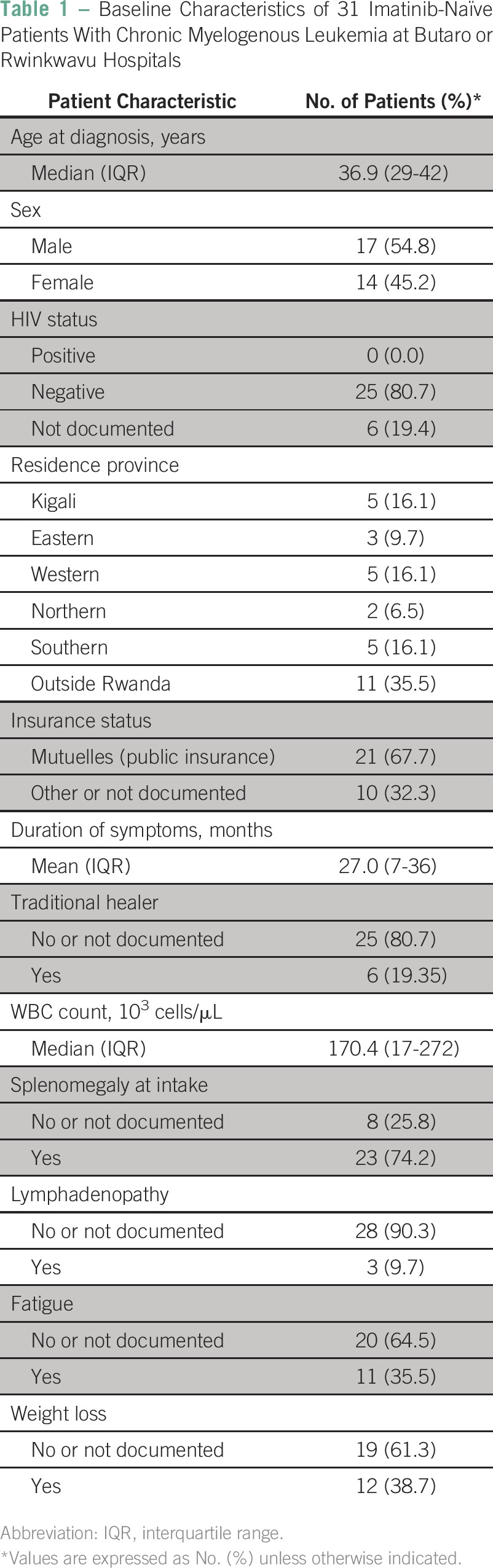
Baseline Characteristics of 31 Imatinib-Naïve Patients With Chronic Myelogenous Leukemia at Butaro or Rwinkwavu Hospitals

### Definitions

The primary outcome for this study was CHR, which was defined according to conventional guidelines as WBC count less than 10,000 cells/μL, absence of splenomegaly on clinical examination, and platelet count less than 450,000 cells/μL. When spleen size was not consistently documented, CHR was replaced with CBC remission, defined as WBC count less than 10,000 cells/μL and platelet count less than 450,000 cells/μL. A secondary outcome was OS, defined as time from imatinib initiation to death from any cause or LTFU. LTFU was defined as having no visit for 9 or more months—the equivalent to three missed visits—before the study end date.

### Ethics

This protocol of this study was approved by institutional review boards in Rwanda (National Health Research Council and Rwanda National Ethics Committee) and the United States (Partners Human Research Committee).

### Statistical Analysis

All analyses were performed using STATA/IC software version 12 (StataCorp, College Station, TX). OS was calculated from the start of imatinib treatment to death, censoring at last follow-up visit for patients who were alive. The Kaplan-Meier product-limit method was used to estimate OS probabilities. An α level of 0.05 was set to determine statistical significance.

## RESULTS

### Baseline Characteristics

A total of 49 patients were treated for CML at Rwinkwavu (n = 25) and Butaro (n = 24) hospitals during the study period. Six patients (12%) were excluded due to incomplete documentation of BCR-ABL status, leaving 43 patients in this cohort (among these, one patient had a single visit). Baseline clinical characteristics and patient demographics are summarized in Table 1, excluding 12 patients who presented to Butaro or Rwinkwavu hospitals having received imatinib before enrollment. Of the 31 imatinib-naïve patients, 17 (54.8%) were men and none were HIV positive. The median age at diagnosis was 36.9 years (interquartile range [IQR]: 29-42 years). Patients presented from all provinces in Rwanda, and 11 (35.5%) patients were from neighboring countries. Two-thirds of patients (n = 21) were enrolled in a public health insurance scheme.

The duration of symptoms before diagnosis ranged from 2 to 144 months (IQR: 4-48 months) among the 23 patients with adequate documentation. Six (19.4%) patients reported having consulted a traditional healer before presenting for care. The most common presenting signs and symptoms, in descending order of predominance, were splenomegaly (74.2%), weight loss (38.7%), and fatigue (35.5%).

### Treatment

Twelve (27.9%) patients had been treated with imatinib before presentation at Rwinkwavu or Butaro hospitals. An additional 12 (27.9%) patients had received other treatment, primarily hydroxyurea, whereas the remaining 19 (44.2%) had never been treated for CML. All patients were initiated or continued on imatinib. (The IQR was 0 to 166 days for the 31 patients who had not previously been on imatinib; of the 12 patients who had previously been on imatinib, all except for one were initiated on imatinib on the day of enrollment). Imatinib dose reduction was instituted for 16 (37.2%) patients, and discontinued for five. Table 2 summarizes reasons for dose reduction, with the leading reasons being isolated thrombocytopenia (31.3%) and neutropenia (31.3%). During the course of study follow-up, reduced doses of imatinib ranged from 100 mg (n = 4, 9.5%), to 200 mg (n = 8, 19.1%), to 300 mg (n = 4, 9.5%). Four (9.5%) patients required dose increases to 600 mg.

**Table 2:**
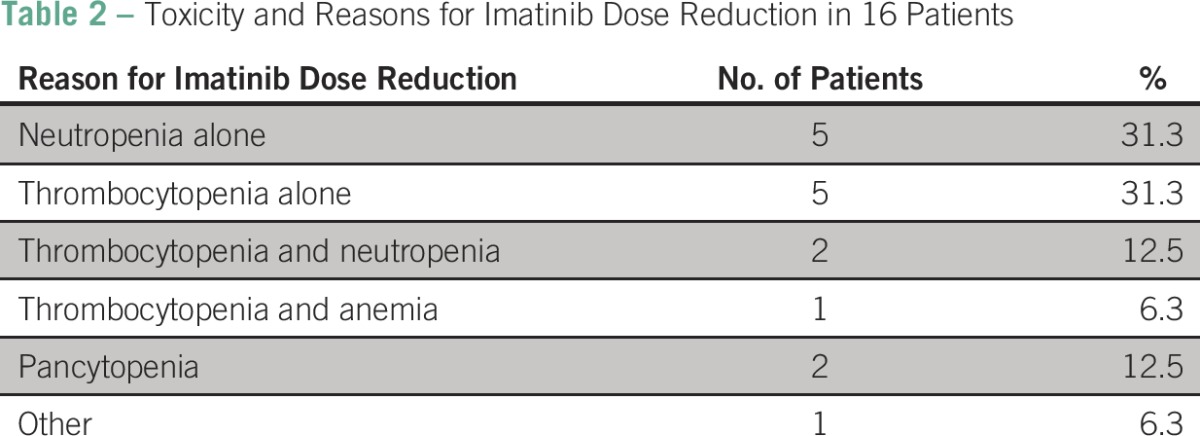
Toxicity and Reasons for Imatinib Dose Reduction in 16 Patients

### Outcomes

Of the 43 patients treated at our centers, 32 (82.1% of those with adequate documentation) remained in CBC remission as of their last evaluation, with a maximum follow-up of 58 months (median follow-up, 22.6 months). Twenty-eight (100% patients with adequate documentation), 27 (90.0%), and 17 (77.3%) patients were in CBC remission at 3, 6, and 12 months of follow-up, respectively. CHR was achieved in 12 (75.0% of patients with adequate documentation), 11 (68.8%), and seven (63.6%) patients, respectively, for the same time points (Table 3).

**Table 3:**
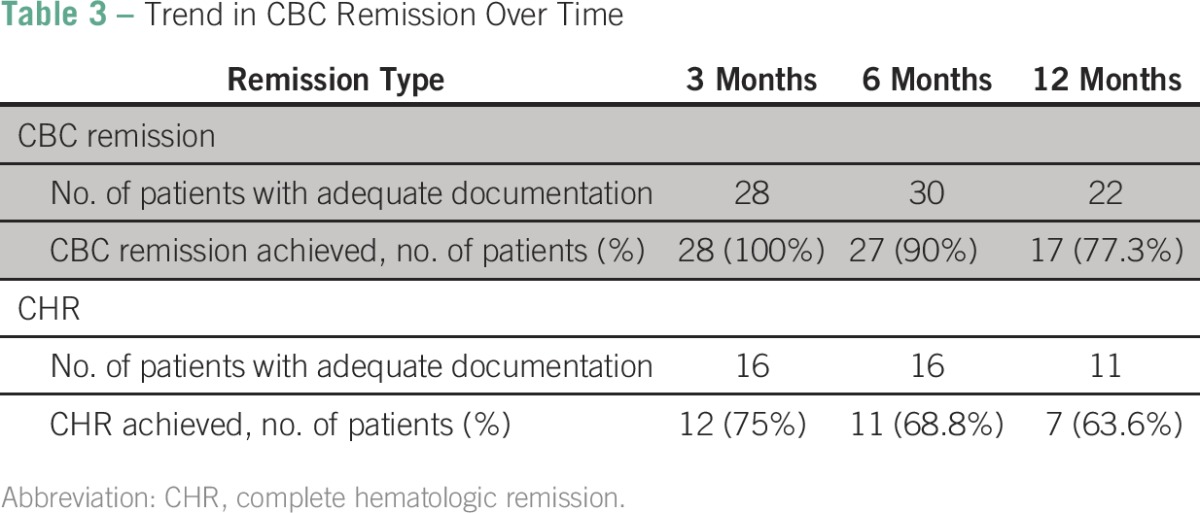
Trend in CBC Remission Over Time

**Fig 1:**
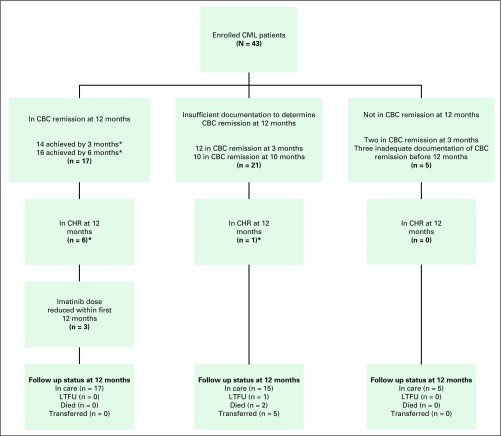
Outcomes at 12 months of follow-up. CHR, complete hematologic remission; CML, chronic myelogenous leukemia; F/u, follow-up; LTFU, lost to follow-up.

Figure 1 summarizes patient outcomes at 12 months of follow-up. Seventeen patients were in CBC remission. Five patients were not in CBC remission (of these, two were in CBC remission at 3 months and subsequently progressed, whereas the remaining three patients had inadequate documentation to assess CBC remission before 12 months). A total of 21 patients did not have adequate documentation to determine CBC remission status; of these, 12 had been in CBC remission at 3 months.

**Fig 2:**
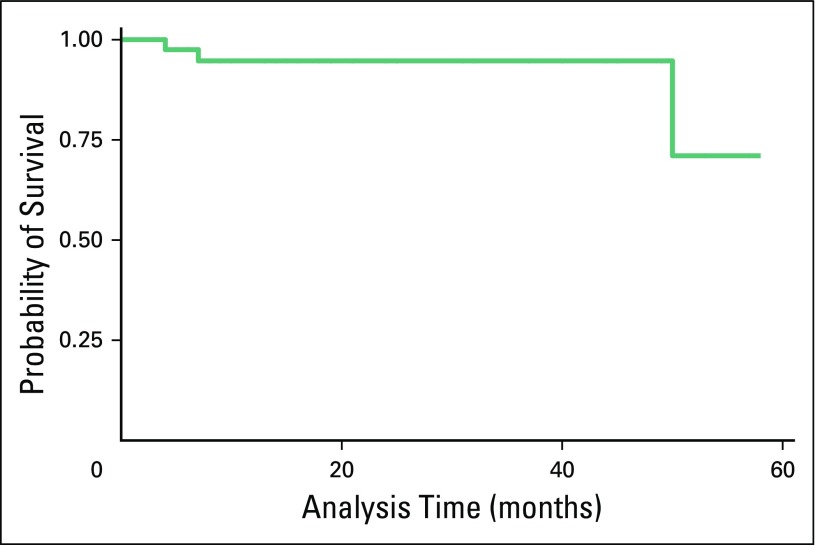
Overall survival among patients with chronic myeloid leukemia managed at Butaro or Rwinkwavu hospitals.

As of June 30, 2014, three patients were known to have died. Two deaths of patients residing outside of Rwanda occurred in the community as a result of unclear causes within 12 months of follow-up. The third death was of a patient admitted to a national referral hospital, who was in presumed blast crisis after being intermittently treated with imatinib for 4 years. Five (11.6%) patients were transferred to other facilities. Four patients were LTFU. The estimated OS at 12 months was 94.7% (95% CI, 0.80 to 0.99); the OS was the same at median follow-up (Fig 2).

## DISCUSSION

In this study, we demonstrate the successful delivery of CML therapy in two rural district hospitals in Rwanda. Most published studies on CML management and outcomes in LMICs describe programs in private and/or urban academic centers.^9-16^ In contrast, our experience is one of the few documented that describes care in a low-income country and serves poor patients in rural areas, with care directed by generalist physicians and nurses. In our cohort, treatment was successful for the majority of patients, with CBC remission exceeding 60% at 3 months of therapy. The CHR at that time was only 28%, although the actual rate of hematologic response was probably higher but difficult to ascertain as a result of poor documentation of spleen size. Treatment was well tolerated, with relatively few dose reductions and no durable treatment stops for toxicity.

Our findings are similar to reports from other LMICs including Sudan, Nigeria, Kenya, Togo, South Africa, Iraq, China, and Mexico, although few of these sites treat patients in settings as remote as Butaro or Rwinkwavu.^6,8-17^ In the largest of these studies, conducted at centers in India, OS at median follow-up of 47 months was 94% and progression-free survival was 76%.^13^ In 2013, Mendizabal et al analyzed the GIPAP database of 33,985 patients treated for CML in Asia, Africa, Latin America, and Southern/Eastern Europe. OS at 3 years was 89.4% (95% CI, 88.9 to 89.9),^17^ identical to the 5-year OS rate in the IRIS study of 89%.^4^ In the same study, Mendizabal et al^17^ demonstrated a lower age at diagnosis of CML in LMICs (37.8 years) when compared with high-income countries (eg, 64.0 years in the United States). This is also consistent with our study findings, where the median age was 36.9 years. Although reasons for this difference have not yet been elucidated, environmental risk factors may be contributing and need to be studied further.^17^

In our cohort of 43 patients, OS at median follow-up of 22.6 months was 94.7% and LTFU was 11.8%. These results are encouraging and demonstrate that CML can be treated successfully in extremely resource-constrained settings, where imatinib is the only available therapeutic intervention and other more complex treatments (eg, alternative tyrosine kinase inhibitors or stem cell transplantation) are not yet available. Furthermore, these outcomes have been attained without the availability of molecular assessment of response to therapy, as is standard in high-resource settings. Monitoring on the basis of only clinical and hematologic assessment was a necessity given the available resources, with neither cytogenetic nor molecular testing available within the country. Additionally, molecular monitoring is costly and had little practical implication given that if resistance to imatinib had developed, other treatment options were not available. Similar constraints have resulted in hematology-based monitoring approaches in Sudan,^10^ with reasonable outcomes (87.5% CHR after 8 weeks of treatment, 16% deaths after 63 months of follow-up in a cohort of 31 pediatric patients). In-country capacity to detect BCR-ABL translocations will soon be available using the Xpert BCR-ABL Monitor (Cepheid, Sunnyvale, CA). Although less expensive than sending specimens abroad, this testing is not without cost and effort. It should be used with the introduction of therapeutic options if molecular relapses are detected.

Our study has several limitations. One is that because of the retrospective design, documentation was not always complete (including spleen size and blood testing), which limited reporting on CHR. In addition, some patients were referred from other facilities with inadequate documentation. Bone marrow biopsies were not performed in all patients as a result of limited resources; hence, we were not able to describe CML phase at presentation or follow-up, nor progression-free survival. The use of consistent bone marrow biopsies is now a priority in the programs at Rwinkwavu and Butaro and is being integrated into routine care. Our study was further limited in its characterization of imatinib-related toxicity. Nonhematologic adverse effects such as nausea, rash, edema, and muscle cramps cited in other studies^4,5,17^ were probably underreported because clinicians may not have actively assessed for these. Finally, given the absence of a universal standardized definition of LTFU for CML, we were not able to compare our rates to those seen in other programs.

Adherence to medication regimens has been shown to be an issue in both resource-rich and resource-constrained settings. A recent United States–based study revealed that high adherence rates were critical to achieving and maintaining major molecular response in CML.^15^ Drug cost, inconvenience, toxicity, and lack of understanding of the importance of compliance all contribute to poor drug adherence. The fact that our patients achieved and maintained hematologic remission suggests that there is an acceptable rate of medication adherence, although formal evaluation of adherence should be performed in the future. Ongoing adherence support initiatives include standardized patient education and telephone check-in.

Approximately one-third of patients required a dose reduction as a result of toxicity, which was monitored with symptom review and hematologic testing performed at follow-up visits. The incidence of thrombocytopenia and neutropenia was similar to that reported in the IRIS study.^4,5^ Importantly, this suggests that imatinib is well tolerated and retains its therapeutic benefit in this setting, despite the expected higher prevalence of malnutrition, malaria, and other comorbidities, and presentation with more long-standing disease among our patients.

Compared with patients in high-income countries, many patients in our setting presented with long-standing disease. Possible reasons for late presentation are multifactorial. Many patients consult traditional healers first (almost one-third reported having done so among our patients), many wait until symptoms are debilitating because of limited funds to travel the long distances required to reach facilities where treatments are available, and many lack awareness of cancer and the potential gravity of illness. Other reasons for delay may reflect limited knowledge about CML of primary care clinicians and operational challenges in diagnostic work up for patients.^17^

A relatively small number of patients were LTFU. Retention supports are particularly important in our setting, given the long duration of follow-up, the socioeconomic vulnerability of most patients, and the long distances that many must travel to access care. Several initiatives exist to support patient follow-up. Missed visits are flagged in the electronic medical records system, oncology nurse coordinators make routine telephone calls to inquire if patients have missed appointments, and transport vouchers and CHW accompaniment are available. We believe that provision of these coordination and socioeconomic supports has been instrumental in achieving high retention among patients with CML. However, follow-up remains a challenge, particularly for patients residing outside the hospitals’ catchment districts, as a result of distance from facilities, frequent changes in cell phone numbers, relocations, and lack of community-based mechanisms to reach patients. All four patients who were LTFU resided outside the hospitals’ catchment districts.

Our findings also highlight the importance of developing strategies to bring high-cost, life-saving treatment to people who do not have the ability to pay. The model that GIPAP and the Max Foundation have developed with Novartis demonstrates the feasibility of doing this safely and effectively. Without GIPAP, imatinib would have been unaffordable for these programs and patients, and all of them would have died early of preventable deaths. We hope this serves as a model for other pharmaceutical companies to develop similar programs to bring their life-saving medications to people in need.

In conclusion, our experience indicates that CML can be effectively managed in a resource-constrained rural setting with promising outcomes, despite limited availability of on-site diagnostic resources or specialty oncology personnel. The simple daily oral regimen and subsidized availability of imatinib make life-prolonging treatment of CML possible in these settings. This was achieved through a public-private partnership designed to transfer knowledge, skills, medications, and technology. Expansion of services in Rwanda is underway, including a nationally approved diagnostic and treatment protocol for CML, a national scale-up of cancer programs, expansion of the imatinib-procurement process through GIPAP, and diversification of in-country testing via Xpert BCR-ABL Monitor.
